# Nanopore sequencing enables tissue-of-origin and pathogen detection in plasma cell-free DNA from critically ill patients

**DOI:** 10.1038/s41420-025-02828-8

**Published:** 2025-10-24

**Authors:** Cyril Willemart, Mojca Strazisar, Tim De Pooter, Tom Stroobants, Thomas Demuyser, Philippe Jorens, Eric Hoste, Gerben Menschaert, Tom Vanden Berghe

**Affiliations:** 1 Cell Death Signaling lab, Infla-Med Centre of Excellence, Department of Biomedical Sciences, University of Antwerp, Antwerp, Belgium; 2 Neuromics Support Facility, VIB Center for Molecular Neurology, Antwerp, Belgium; 3 Department of Biomedical Sciences, University of Antwerp, Antwerp, Belgium; 4 Department of Intensive Care Medicine and Clinical Pharmacology (Antwerp University Hospital), Antwerp, Belgium; 5 Laboratory of Experimental Medicine and Pediatrics, Infla-Med Centre of Excellence, University of Antwerp, Antwerp, Belgium; 6 Department of Medical Microbiology (Antwerp University Hospital), Antwerp, Belgium; 7 AIMS Lab, Center for Neuroscience, Vrije Universiteit Brussel, Brussels, Belgium; 8 Laboratory of Applied Microbiology and Biotechnology (LAMB) University of Antwerp, Antwerp, Belgium; 9 Intensive Care Unit, Department of Internal Medicine and Pediatrics, Ghent University Hospital, Ghent, Belgium; 10 Department of Mathematical Modelling, Statistics and Bioinformatics, Ghent University, Ghent, Belgium; 11 Department of Biomedical Molecular Biology, Ghent University, Ghent, Belgium

**Keywords:** Diagnostic markers, Epigenetics, Infection

**To the Editors**,

In the intensive care unit (ICU), organ injury and infection are leading causes of mortality [1]. Both processes release cell-free DNA (cfDNA) into the bloodstream: injured tissues release fragments that retain methylation marks for tissue-of-origin inference, and—when infection is present—pathogens contribute microbial cfDNA [2–5]. Here, we report that low-coverage Oxford Nanopore Technologies (ONT) sequencing of plasma cfDNA captures both signals in a single assay, enabling simultaneous tissue-injury and pathogen readouts in critically ill patients. In 44 samples from 34 patients (median coverage ~0.8X; methods in Supplementary Methods), we observed biologically consistent tissue signals and case-level microbial concordance. Although previous works reported that ONT cfDNA recovered tissue signals in cancer and viral hepatitis [6, 7], our study is, to our knowledge, the first to demonstrate dual host–pathogen diagnostics in a heterogeneous ICU cohort at a bedside-relevant sequencing depth.

Plasma cfDNA concentration was significantly higher in samples from patients who did not survive the ICU stay compared with survivors (Fig. 1A) and correlated with total Sequential Organ Failure Assessment (SOFA) score (Fig. 1B). Hepatocyte-derived cfDNA correlated with alanine aminotransferase (ALT) (Fig. 1C); cardiac-derived cfDNA with troponin T (Fig. 1D); and lung-derived cfDNA with PaO_2_/FiO_2_ (Fig. 1E). No correlation was observed between kidney-derived cfDNA proportion and urinary chitinase-3-like protein 1 (u-CHI3L1) (Fig. 1F). T-cell-derived cfDNA proportion was significantly elevated in patients with suspected infection (Fig. 1G). Together, these results suggest that low-coverage ONT cfDNA methylation profiling provides biologically meaningful insights into organ-specific injury and immune activation in critically ill patients. These tissue-specific associations align with prior work using alternative methods: lung-derived cfDNA correlates with COVID-19 severity [8], cardiac-derived cfDNA correlates with troponin in surgery and myocardial infarction [9, 10], and hepatocyte-derived cfDNA tracks ALT across transplantation, sepsis, and COVID-19 [3, 5, 8, 11]. Conversely, kidney-derived cfDNA has repeatedly shown poor correlation with functional markers [12], aligning with our observations.

**Fig. 1:**
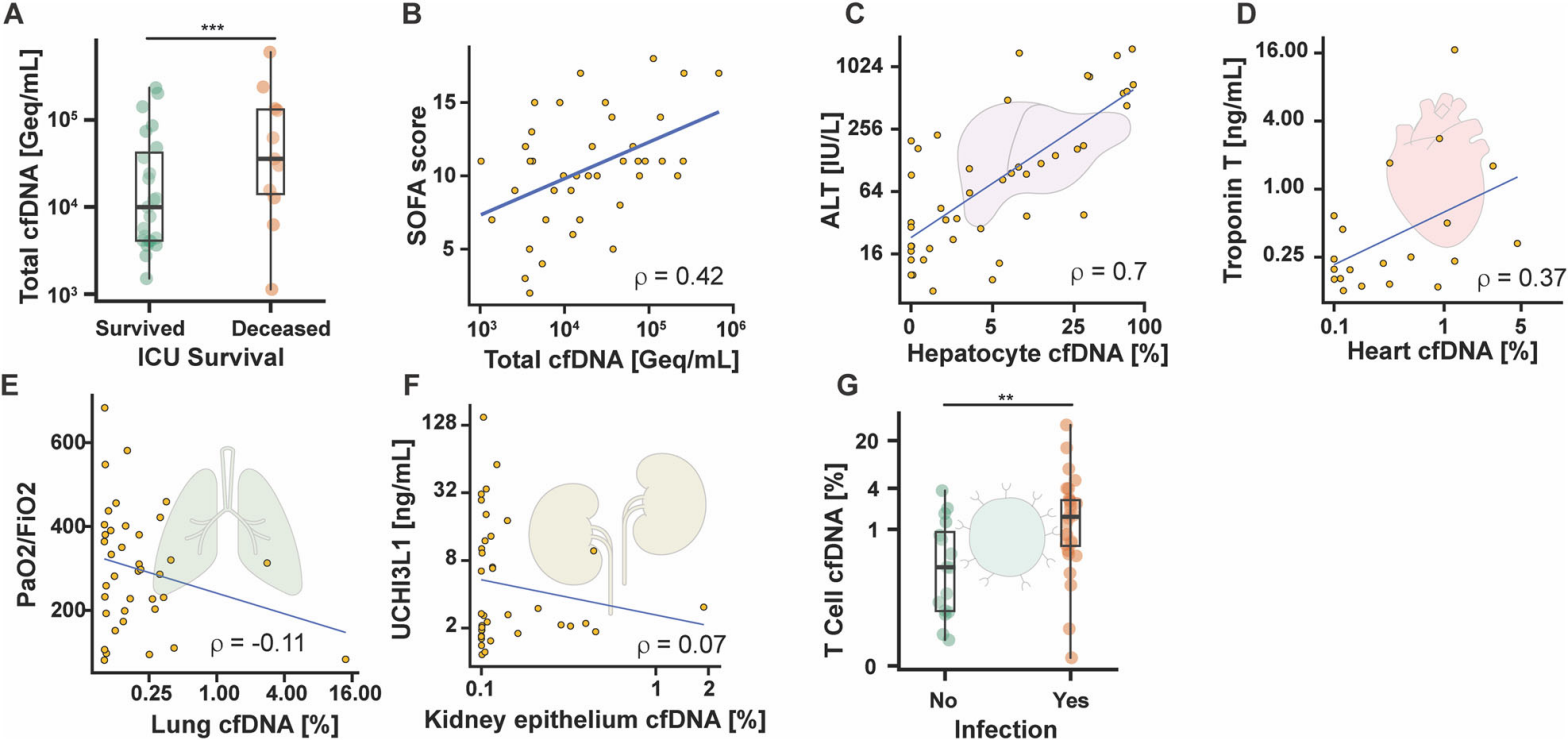
Comparison of cfDNA deconvolution results with clinical data. **A** Plasma cfDNA concentrations in patients who survived in the ICU vs. those who did not survive. **B** Total SOFA score vs. plasma cfDNA concentrations. **C** ALT vs. proportion of hepatocyte-derived cfDNA. **D** Troponin T vs. proportion of heart-derived cfDNA. **E** PaO_2_/FiO_2_ vs. proportion of lung-derived cfDNA. **F** u-CHI3L1 vs. proportion of kidney-derived cfDNA. **G** Proportion of T-cell-derived cfDNA vs. infection. Comparisons between two groups were performed using the Wilcoxon rank-sum test. *p ≤ 0.05, **p ≤ 0.01, ***p ≤ 0.001. Spearman’s correlation coefficients are depicted. ICU Intensive Care Unit, SOFA sequential organ failure assessment, ALT alanine transaminase, u-CHI3L1 urinary chitinase-3-like protein 1.

Despite these encouraging findings, several factors likely limited tissue deconvolution performance. First, low coverage reduces the sensitivity of low-abundance tissues. Simulations indicated that at 0.5X coverage, a tissue had to contribute >10% of total cfDNA to be detected in >75% of the runs (Fig. S1). Based on these simulations, a minimum coverage of 5X appears necessary to reliably detect tissues present in lower abundances. This could be achieved by reducing the number of pooled samples per flow cell or by using multiple flow cells in parallel. Notably, cfDNA sequencing with current ONT chemistry often yields lower output than genomic DNA, possibly due to pore exhaustion from the shorter fragment lengths characteristic of cfDNA. Second, marker specificity may limit detection sensitivity. Simulations showed that spike-ins of certain tissues, such as the kidney, required larger sample sizes for successful detection, pointing to variability in marker specificity. Third, biological and kinetic factors may affect cfDNA availability: for instance, renal cfDNA may be preferentially excreted into urine, reducing its representation in plasma.

Microbial profiling showed partial concordance with clinical microbiology. Among 30 samples from patients with ongoing infection, microbial DNA was detected in 17, yielding a sensitivity of 57%. Of the 15 infection-negative samples (including 1 healthy control), microbial DNA was correctly absent in 11, corresponding to a specificity of 73%. Several cases showed strong concordance. *Escherichia coli* was detected in four samples, three of which matched clinical cultures. In one patient with pneumonia, *Staphylococcus lugdunensis* was detected in two plasma samples, consistent with hemoculture results. Notably, cfDNA evidence was present at ICU admission, whereas the hemoculture turned positive only on day 3, underscoring the potential for earlier pathogen detection via cfDNA metagenomics. *Nakaseomyces glabratus* and *Klebsiella pneumoniae* were both identified in pneumonia cases, matching a pleural effusion and tracheal aspirate culture, respectively. *Klebsiella michiganensis* was detected where clinical reports described *Klebsiella oxytoca/Raoultella ornithinolytica*, possibly reflecting species misclassification within the *K. oxytoca* complex. *Aspergillus fumigatus* was identified in a sample from a patient diagnosed with pneumonia, where antifungal therapy had been initiated. All microbial hits are listed in Supplementary Table S1.

Important challenges remain for microbial detection. First, non-sterile plasma processing likely introduced environmental background, shared across samples—including a healthy control—consistent with prior reports that many microbial signals in healthy plasma originate from contaminants [13]. To mitigate this, we applied a stringent read threshold to suppress false positives, which excluded some true pathogens at low abundance, such as clinically confirmed *Candida albicans* that fell below the inclusion cut-off. Adaptive, species-aware thresholds combined with pre-test probabilities could improve sensitivity while maintaining precision. Second, sensitivity may have been underestimated by sample-type mismatch: cfDNA was sequenced from plasma, whereas clinical validation relied on other sources such as tracheal aspirates, limiting direct comparison. Third, unexpected hits such as *Enterococcus faecium*, which lacked supporting clinical data, underscore the need for orthogonal validation (e.g., qPCR, systematic cultures) in future studies. Lastly, deeper sequencing would improve detection of rare taxa and enable resistance-gene profiling, both critical for clinical deployment.

In summary, our study establishes that low-coverage ONT sequencing can capture valuable tissue-of-origin and microbial signals from plasma cfDNA in critically ill patients. For accurate detection of low-abundance tissues and less abundant microbial species, a minimum coverage of 5X might be required. While significant technical limitations, including sequencing depth, microbial contamination, and the need for rigorous orthogonal validation, must be addressed, our results provide a foundation for developing scalable, real-time diagnostics in intensive care. Future research should validate these findings in larger patient cohorts, implement longitudinal tracking to assess cfDNA’s utility for dynamic risk stratification, and optimize methods to enhance sensitivity and specificity for both host and microbial targets, thereby enabling personalized clinical decision-making.

## Supplementary information

Supplementary Figures and Methods

Supplementary Table S1: Normalized microbial cfDNA reads per samples and comparison with clinical infection data.

## Data Availability

All analysis scripts and code used in this study are publicly available on GitHub at https://github.com/tramelliwe/cfDNA_CDDis.
