# Supplementary material for: Nanopore sequencing enables tissue-of-origin and pathogen detection in plasma cell-free DNA from critically ill patients
Source: Cell Death Discov. 2025 Oct 24;11:484. doi: 10.1038/s41420-025-02828-8 (PMC12552604; doi:10.1038/s41420-025-02828-8)
Supplement: Supplementary file 1 — Supplementary Figures and Methods [file 41420_2025_2828_MOESM1_ESM.docx]

**Nanopore Sequencing Enables Tissue-of-Origin and Pathogen Detection in ICU Plasma cfDNA**

Cyril Willemart, Mojca Strazisar, Tim De Pooter, Tom Stroobants, Thomas Demuyser, Phillipe Jorens, Eric Hoste, Gerben Menschaert, and Tom Vanden Berghe

Table of Contents

[Supplementary Methods 2](#_Toc208336105)

[Supplementary Figures 3](#_Toc208336106)

# Supplementary Methods

We collected 44 plasma samples (500 µL) from 34 ICU patients and extracted cfDNA using the Sera-Xtracta™ Cell-Free DNA Kit (Cytiva). DNA repair was performed with the NEBNext FFPE DNA Repair v2 Module (New England Biolabs), followed by library preparation according to ONT’s standard protocols. Libraries were sequenced on PromethION R10.4.1 flow cells. A lambda phage DNA spike-in was added to the samples at 0.1% molar ratio as an internal control for normalization and quality assessment during nanopore sequencing. Reads were basecalled and aligned to the human reference genome (hg38) using *minimap2*, with an median genome-wide coverage of ~0.8× (range 0.3-1.5). DNA methylation was profiled using *modkit*.

Tissue-of-origin deconvolution was based on tissue-specific methylation markers derived from publicly available WGBS data (GEO GSE186458). The genome was segmented into regions of consistent methylation (≥3 CpGs, ≤1,000 CpGs, ≤2,000 bp), and the top 500 differentially methylated regions (DMRs) per tissue were selected using *wgbstools* [1]. Markers lacking tissue specificity were removed, yielding ~16,000 DMRs with an average length of 250 bp. We applied the *CelFiE* deconvolution algorithm using default parameters and two unknown tissues [2]. *CelFiE* was chosen for its robust performance and correlation with clinical biomarkers.

To evaluate deconvolution accuracy, we created in silico cfDNA mixtures from the same WGBS data using a leave-one-out framework. One sample per tissue was held out as a test set, and tissue-specific markers were trained on the remainder. Reads were sampled at defined proportions and coverages, and deconvolution was performed using *UXM*, a high-speed method with comparable accuracy to *CelFiE*, enabling large-scale benchmarking [3].

Microbial analysis was performed on non-human reads using *Kraken2* with a custom database [4]. Reads were quality-filtered, and likely contaminants were removed through k-mer-based and empirical filtering strategies, including exclusion of taxa with batch-specific patterns or low absolute abundance. Infection was diagnosed through standard-of-care clinical work-up.

The Sequential Organ Failure Assessment (SOFA) score was calculated daily using the worst levels of platelets count, PaO2/FiO2 ratio, creatinine level, urinary output, bilirubin level, arterial tensions, and Glasgow Coma Scale [5].

[1] N. Loyfer, J. Rosenski, and T. Kaplan, “wgbstools: A computational suite for DNA methylation sequencing data representation, visualization, and analysis,” May 10, 2024, *Bioinformatics*. doi: 10.1101/2024.05.08.593132.

[2] C. Caggiano *et al.*, “Comprehensive cell type decomposition of circulating cell-free DNA with CelFiE,” *Nat Commun*, vol. 12, no. 1, p. 2717, May 2021, doi: 10.1038/s41467-021-22901-x.

[3] N. Loyfer *et al.*, “A DNA methylation atlas of normal human cell types,” *Nature*, vol. 613, no. 7943, pp. 355–364, Jan. 2023, doi: 10.1038/s41586-022-05580-6.

[4] D. E. Wood, J. Lu, and B. Langmead, “Improved metagenomic analysis with Kraken 2,” *Genome Biology*, vol. 20, no. 1, p. 257, Nov. 2019, doi: 10.1186/s13059-019-1891-0.

[5] J.-L. Vincent *et al.*, “The SOFA (Sepsis-related Organ Failure Assessment) score to describe organ dysfunction/failure: On behalf of the Working Group on Sepsis-Related Problems of the European Society of Intensive Care Medicine (see contributors to the project in the appendix),” *Intensive Care Med*, vol. 22, no. 7, pp. 707–710, Jul. 1996, doi: 10.1007/BF01709751.

#
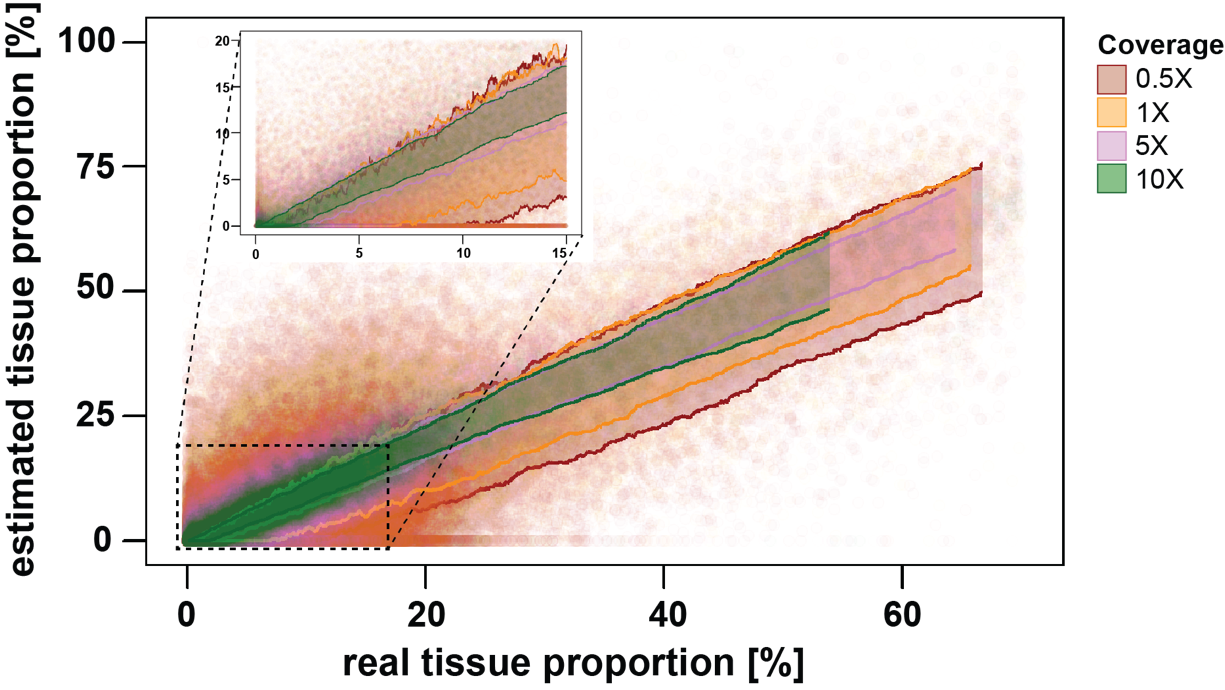
Supplementary Figures

**Figure S1: Deconvolution of simulated cfDNA mixtures at different coverages.**
